# Modulated nanowire scaffold for highly efficient differentiation of mesenchymal stem cells

**DOI:** 10.1186/s12951-022-01488-5

**Published:** 2022-06-16

**Authors:** Jose E. Perez, Bashaer Bajaber, Nouf Alsharif, Aldo I. Martínez-Banderas, Niketan Patel, Ainur Sharip, Enzo Di Fabrizio, Jasmeen Merzaban, Jürgen Kosel

**Affiliations:** 1 Bioscience Program, Biological and Environmental Science and Engineering Division, King Abdullah University of Science and Technology (KAUST), Thuwal, 23955-6900 Kingdom of Saudi Arabia; 2 Electrical and Computer Engineering Program, Computer, Electrical and Mathematical Science and Engineering Division, King Abdullah University of Science and Technology, Thuwal, 23955-6900 Kingdom of Saudi Arabia; 3 Dipartimento di Scienza Applicata e Tecnologia, Politecnico di Torino, Corso Duca Degli Abruzzi 24, 10129 Turin, Italy; 4 Division of Sensor Systems, Silicon Austria Labs, High Tech Campus Villach, 9524 Villach, Austria

**Keywords:** Stem cells, Cell scaffold, Nano surface, Magnetic field, Osteogenesis, Nanowires, Magnetic materials

## Abstract

**Background:**

Nanotopographical cues play a critical role as drivers of mesenchymal stem cell differentiation. Nanowire scaffolds, in this regard, provide unique and adaptable nanostructured surfaces with focal points for adhesion and with elastic properties determined by nanowire stiffness.

**Results:**

We show that a scaffold of nanowires, which are remotely actuated by a magnetic field, mechanically stimulates mesenchymal stem cells. Osteopontin, a marker of osteogenesis onset, was expressed after cells were cultured for 1 week on top of the scaffold. Applying a magnetic field significantly boosted differentiation due to mechanical stimulation of the cells by the active deflection of the nanowire tips. The onset of differentiation was reduced to 2 days of culture based on the upregulation of several osteogenesis markers. Moreover, this was observed in the absence of any external differentiation factors.

**Conclusions:**

The magneto-mechanically modulated nanosurface enhanced the osteogenic differentiation capabilities of mesenchymal stem cells, and it provides a customizable tool for stem cell research and tissue engineering.

**Graphical Abstract:**

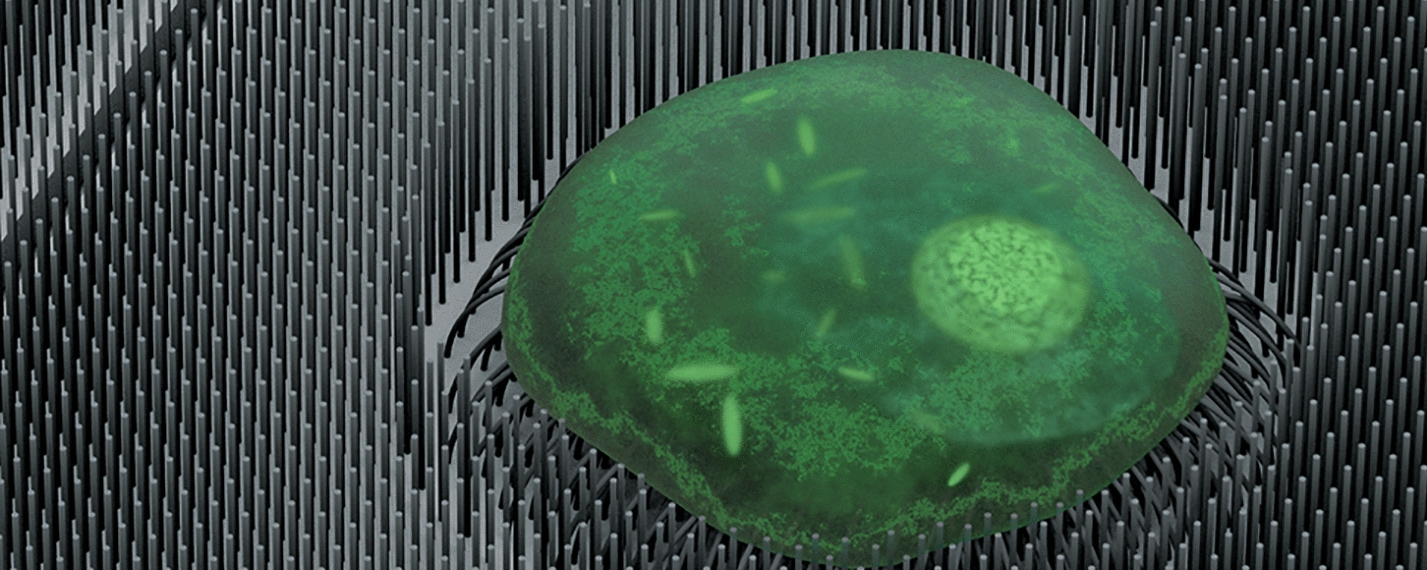

**Supplementary Information:**

The online version contains supplementary material available at 10.1186/s12951-022-01488-5.

## Background

The use of stem cells is becoming increasingly attractive in cell-based tissue engineering and regeneration research. In particular, transplanted mesenchymal stem cells (MSCs) can directly participate in bone formation by differentiating into osteoblasts that generate mineralized tissue resembling bone capable of rehabilitating and improving bone regeneration [1–4]. Current experimental bone tissue engineering protocols utilize a combination of biochemical factors that promote the expression of various osteogenic markers such as runt-related transcription factor 2 (Runx2), osteopontin (OPN), osteocalcin (OCN), type 1 collagen, and alkaline phosphatase (ALP) [5]. However, factors related to long treatment duration, side effects, variability, and cost limit their usability. New strategies are being devised to overcome these issues by exploiting the role played by extracellular stimuli on cell fate [6]. Matrix stiffness [7], as well the nano/micro-scale geometry [8, 9] and its influence on cell morphology [10] are major factors contributing to stem cell fate.

Current progress in nano-engineering techniques has advanced the use of biomaterials as cell matrices or scaffolds to study the effects of physical cues on MSCs as an alternative strategy in bone tissue engineering therapy to increase bone regeneration [11, 12]. Furthermore, biomaterial scaffolds emphasize nanotopographical cues that recapitulate the interactions between cells and the extracellular matrix (ECM) via integrin receptors to enhance their growth and guide their fate [13]. For instance, the particular cues provided by nanopits [14, 15], nanoarrays [16] and spatially discrete patterned surfaces [17] influence stem cell fate. Similarly, dense nanowire (NW) arrays provide a unique and adaptable nanotopograhy that can drive mesenchymal stem cell differentiation into osteoblasts [18], and human induced pluripotent stem cells into neurons [19].

Recently, the effects of additional external stimuli on culture scaffolds and translation into stem cell fate regulation were evaluated. Stimuli such as electrical impulses, ultrasound waves and magnetic fields were studied in the context of stem cell fate control [20]. Among these, magnetic fields are of special interest due to their inherent targetability and non-invasiveness in vivo. Moreover, magnetically-responsive scaffolds show a synergistic stem cell fate control with that observed solely by static [21] and/or pulsed [22] electromagnetic field application. For instance, the combined effect of a pulsed electromagnetic field and osteogenic medium on MSCs grown on a polycaprolactone nanofibrous scaffold enhances the expression of OCN, Runx2, ALP and type 1 collagen osteogenic markers after 1 week of culture [23]. This synergy is improved following addition of magnetic nanoparticles into the polycaprolactone matrix and induces bone formation after 6 weeks of static field application in vivo [24].

We previously reported that dense, vertically aligned Fe NWs induce cytoskeletal changes in MSCs [25]. Here, we couple a magnetic field to a magnetoreceptive nanotopography (an array of NWs) to modulate the nanosurface. The magnetic field caused bending of the NWs, or a deflection at the free ends of their tips. Given the important role of physical cues and mechanotransduction in guiding stem cell differentiation, we hypothesized that in addition to the cues provided by the NW scaffold a mechanical stimulus induced by the magnetically actuated NWs enhances the osteogenic differentiation. We investigated the expression of the early osteogenic marker OPN, as well as that of OCN, Runx2 and ALP, in MSCs cultured on the NW scaffold. Specifically, the aim was to show whether the application of a magnetic field to the magnetoreceptive NW scaffold would increase the expression of these markers in comparison with the effects of the nanotopography by itself.

## Results and discussion

### Osteogenic differentiation of mesenchymal stem cells cultured on magnetic nanowires

**Fig. 1:**
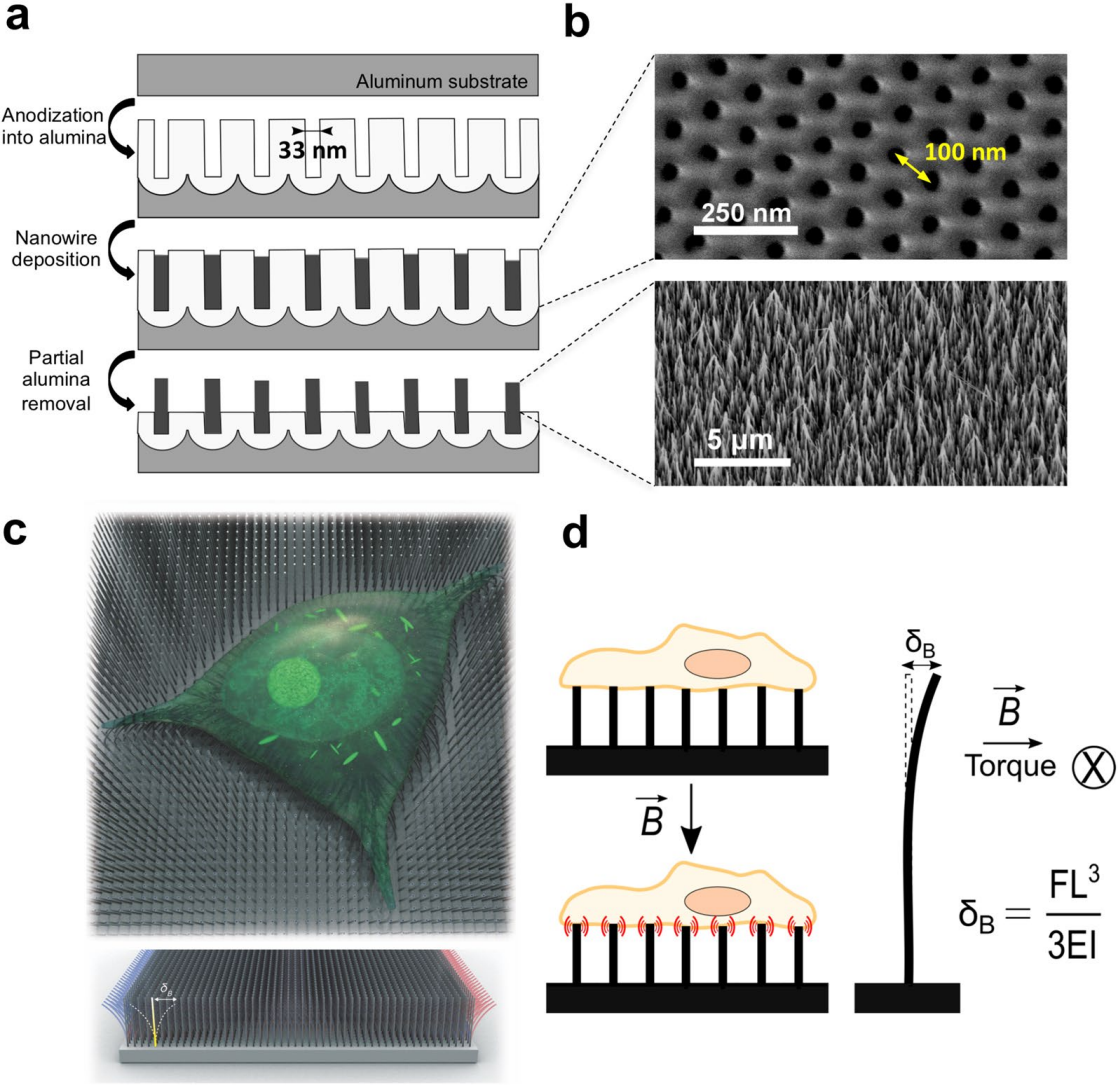
Magnetically modulated nanosurface. **a** Nanoporous template fabrication and NW electrodeposition process. **b** Scanning electron microscopy images of the nanoporous alumina template (top view) and of the NW scaffold after the partial removal of the alumina. The average pore (or NW) diameter is of 33 nm, and the average spacing between each of them is of 100 nm. **c** Concept design of a cell cultured on top of the NW scaffold. **d** The free end of the NW experiences an elastic deflection *δ*_*B*_ when under a magnetic field *B*. *F* = force applied to the system, *L* = length of the NW, *E* = elastic modulus, *I* = moment of inertia.

**Fig. 2:**
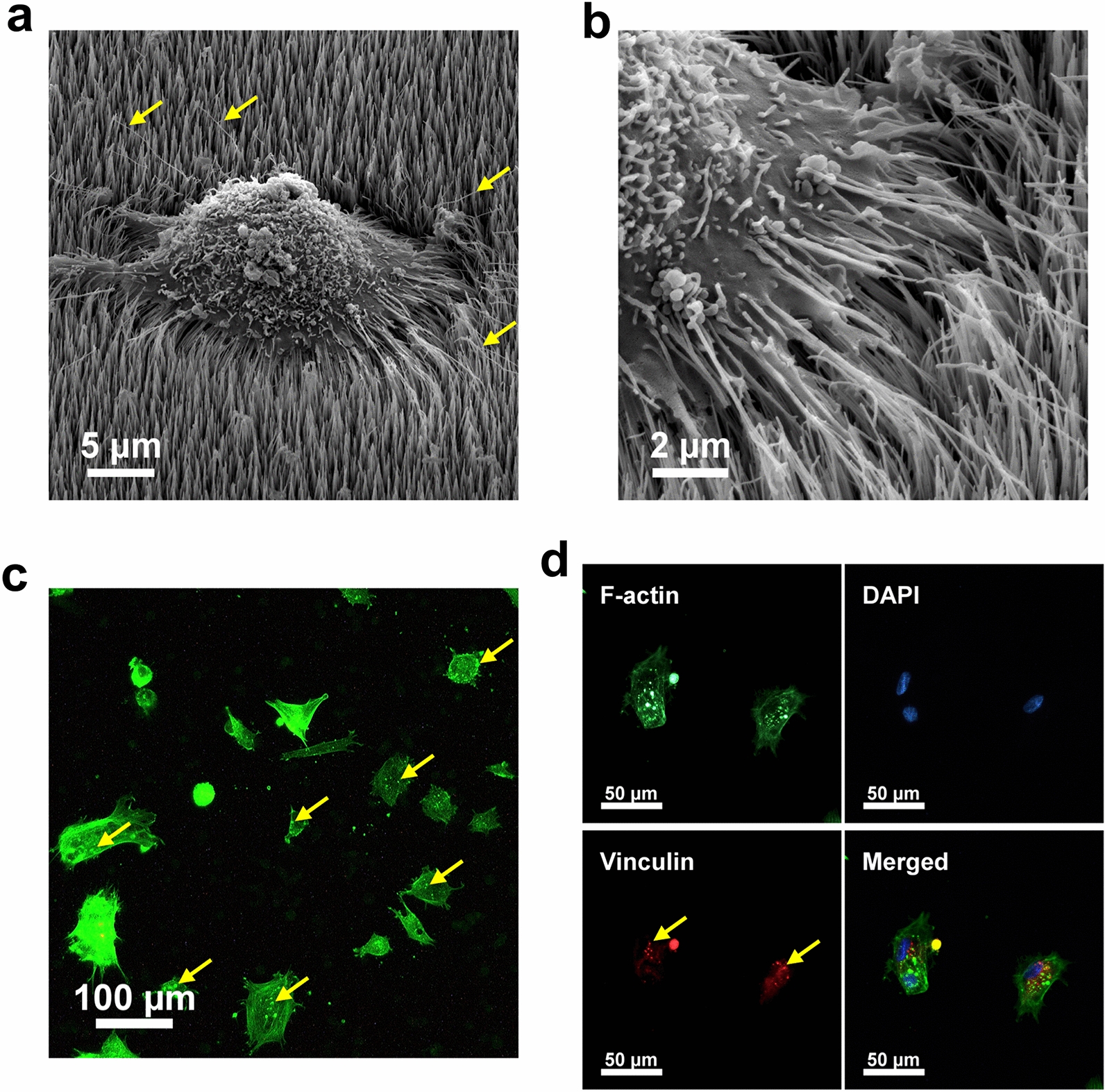
MSCs adopt a contracted shape after 2 days of culture. Scanning electron micrographs of MSCs cultured on the NW scaffold (**a**), with focal adhesion points forming around the NWs (**b**). Representative images of approximately 20 cells. **c** F-actin fluorescence staining of MSCs on the NW scaffold. The cell area is of 1628 ± 145 µm^2^, as determined by F-actin staining (*n* = 50 cells). **d** Immunofluorescence staining of the focal adhesion protein vinculin. Arrows in **a** denote filopodia around the cell periphery; arrows in **c** and **d** denote the aggregation of actin and vinculin in highly localized spots, respectively.

The fabrication process of the biocompatible NW scaffold was previously described in detail [25]. Briefly, an aluminum substrate is anodized in order to create a nanoporous alumina template, into which the NWs are electrodeposited (Fig. 1a). The diameter of the pores and the spacing between each of them define the average NW diameter and the spacing between each NW, respectively, after the electrodeposition process (Fig. 1b). Partial removal of the alumina template through a wet etching process reveals a free-standing network of NWs partially embedded in the template (Fig. 1b). For our experimental fabrication conditions, the template yields NWs with a length of 2–3 µm, an average diameter of 33 nm and an inter-NW spacing of 100 nm. Figure 1c and d show the concept of the magnetic field-modulated nanosurface, on top of which cells are cultured. When a magnetic NW is exposed to a magnetic field *B*, a torque is generated as the magnetic moment along the axis of the NW aligns to the direction of the magnetic field. The elastic deflection experienced by the tip of each NW was approximately *δ*_*B*_ = 100 nm using an an end-loaded cantilever beam model after estimating the unloaded deflection *δ*_*B*_ at the free end of the NW due to the magnetic torque (Additional file 1). If the deflection was blocked, the maximum force exerted by the NW to the cell was around 240 pN. Figure 2a shows a single, contracted MSC on the NWs after 2 days of culture; this shape was adopted due to the scaffold nanotopography. The cell can be observed actively probing the NW surface using filopodia, which are actin-rich membrane projections used to establish adhesion [26]. The formation of multiple focal adhesion points around the NWs caused the NWs to bend as the cell attached and grew on the scaffold (Fig. 2b). This was confirmed by the contracted expression of the cytoskeleton protein F-actin (Fig. 2c) and the highly localized expression of the focal adhesion protein vinculin (Fig. 2d). Using the average cell area around 2 days of culture on the scaffold of 1628 ± 145 µm^2^ (Fig. 2c), as well as the nanowire spacing of 100 nm (Fig. 1b), we can estimate the approximate number of NWs interfaced per cell (the NW dose) at around 165,000 NWs/cell.

Cell shape and contractility are mainly driven by stress fibers composed of bundles of actin and myosin [27]. The adoption of a contracted cell shape is thus likely due to the restructuring of the actin cytoskeleton as the cell comes into contact with the nanotopography and finds its adhesion points on and around the NWs. The expression pattern of the focal adhesion protein vinculin in localized spots correlates with that of F-actin, lending support to this claim. As one of vinculin’s functions is to anchor F-actin to the cell membrane [28], the observed cell restructuring into a contracted shape could be explained by an interplay between these two cytoskeletal proteins as focal adhesion points are formed.

**Fig. 3:**
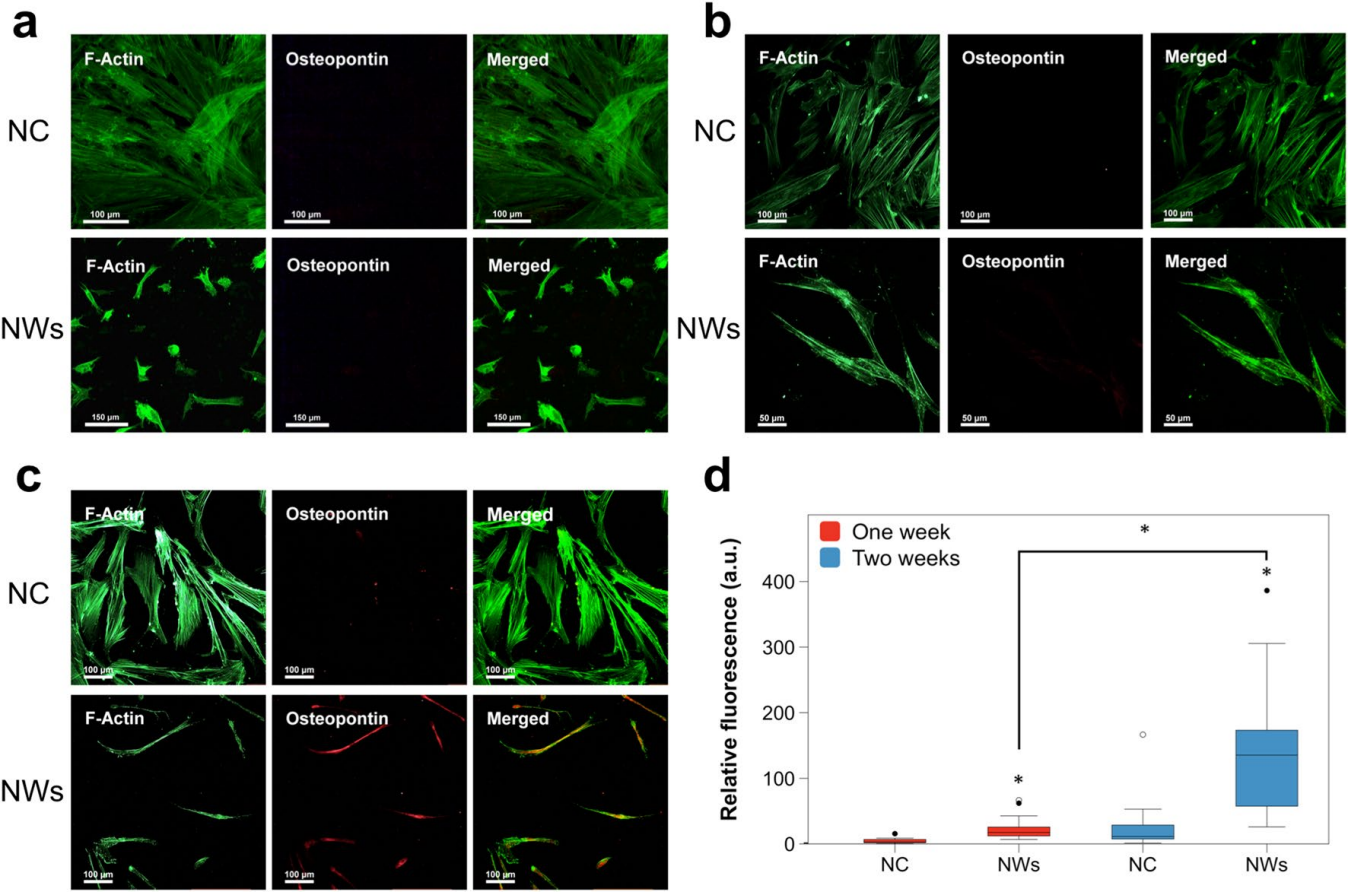
Immunofluorescence staining of osteopontin in MSCs cultured on the NW scaffold. MSCs were cultured for **a** 2 days, **b** 1 week or **c** 2 weeks and then stained for F-actin (green) and OPN osteogenic marker (red). Representative images of *n* = *2* independent experiments. **d** Box plots showing the corrected total cell fluorescence quantification of OPN following the culture of MSCs on the NW surface after 1 and 2 weeks. *n* = 28 imaged cells, *p < 0.01. *NC *negative control (cells cultured on a regular tissue culture-treated plastic).

Next, we investigated whether the shown interface of the MSCs with the NW scaffold topography would lead to the expression of osteogenic markers. For this, an initial focus was put on the ECM protein OPN as an early marker of osteogenesis onset [29]. There was no OPN immunofluorescence signal from MSCs cultured on the NWs after 2 days of culture (Fig. 3a); however, a small increase in immunofluorescence was observed after 1 week (Fig. 3b), and significantly higher immunofluorescence after 2 weeks (Fig. 3c). Quantification of OPN fluorescence revealed significant expression of this protein for both these later culture time points (Fig. 3d). The expression of stem cell markers CD105 and CD73 [30] was observed after 2 days of culture on the NWs by immunofluorescence staining (Additional file 1: Figure S1); however, CD73 expression diminished after 1 week. The overall expression of both markers was reduced compared to the negative control. This suggests that MSCs retain these stem cell markers after 2 days of culture on the NWs; however, their expression decreases with time. Overall, these data are in agreement with the onset of expression of OPN, indicating a change in cell phenotype. It should additionally be noted that the cell shape progresses from the previously observed contracted shape (Fig. 2c) to a more extended and elongated one at the later reported culture time points of 1 and 2 weeks, as seen by F-actin staining (Fig. 3).

Focal adhesions are multi-protein complexes that serve as a link between the cell cytoskeleton and the ECM or substrate. They play a critical role in cell mechanosensing, particularly through the coupling between integrins, which are the main ECM receptors, and the cell cytoskeleton protein F-actin [31]. For instance, the upregulation of integrins has been recently shown to be a driver of osteogenesis by promoting the expression of markers such as Runx2, ALP, and type 1 collagen [32]. Indeed, several integrins were found to be upregulated in MSCs cultured on NWs with a similar nanotopography, along with equal cytoskeleton remodeling and cell shape contraction responses of F-actin and vinculin [18]. In the same vein, it can be expected that the increase in the expression of OPN that we observe here is linked to changes in the cell cytoskeleton and cell adhesion, such as a differential F-actin and vinculin expression, imposed by the probing of the NW surface through integrins and resulting in turn in the activation of mechanosensitive pathways linked to osteogenesis.

### Nanowire surface modulation

**Fig. 4:**
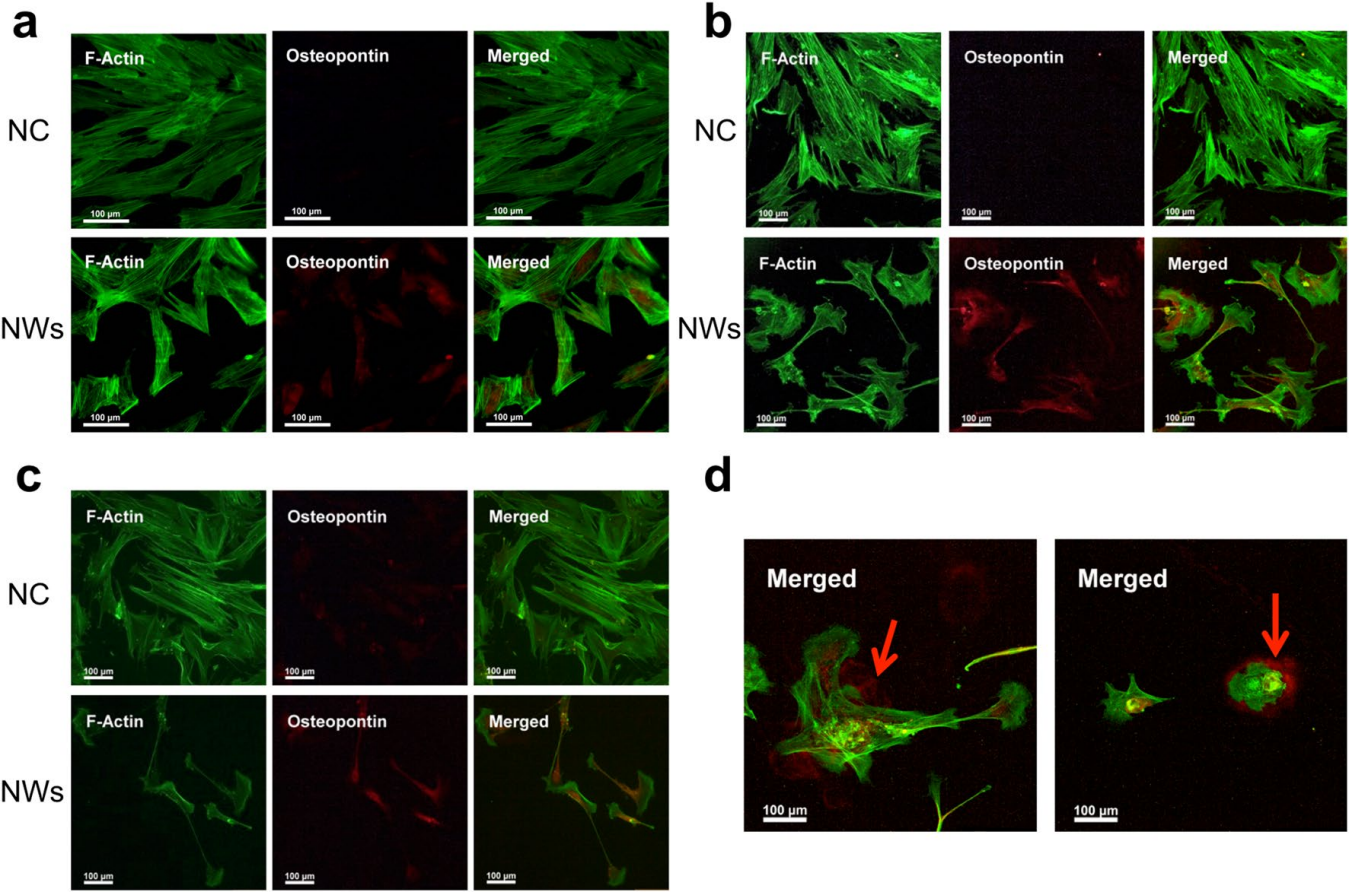
Osteopontin staining in MSCs cultured on NWs exposed to a magnetic field for various durations of time. MSCs were cultured for **a** 2 days, **b** 1 week, or **c** 2 weeks, and a magnetic field with an intensity of 250 mT and a frequency of 0.1 Hz was applied 12 h per day (i.e., 12 h with magnetic field and 12 h without magnetic field). The cells were then stained for F-actin (green) and OPN (red). *NC *negative control. **d** Extracellular OPN expression after 1 week (left) and 2 weeks (right) exposure to a magnetic field under the previously mentioned parameters. Representative images of *n* = 2 independent experiments.

In order to evaluate whether the modulation of the NWs in the scaffold could elicit an additive upregulation of OPN, a low frequency (0.1 Hz), 250 mT magnetic field was applied for 12 h per day to MSCs cultured on the NW scaffold. Immunostaining of OPN protein expression was determined after 2 days, 1 week, and 2 weeks of culture under these magnetic field parameters. This treatment significantly influenced the scaffold to induce OPN expression in MSCs (Fig. 4) compared to the MSCs cultured on non-magnetically activated NWs (Fig. 3). Remarkably, OPN expression was observed after 2 days with the magnetic field (Fig. 4a). The stem cell marker CD105 was only expressed after 2 days of incubation and was quickly lost after 1 week, whereas CD73 expression was absent at all time points (Additional file 1: Figure S2). OPN is a cell-ECM interface structural protein [33], so it is expected to be located outside of the cell body at a certain times during differentiation. Indeed, OPN staining showed that it was extracellular at both the 1 week and 2 week time points tested when MSCs were cultured on magnetically activated NWs (Fig. 4d).

As mentioned previously, nanotopographical cues drive the osteogenic differentiation in MSCs through changes in cell adhesion and cytoskeletal intracellular tension, particularly through the lengthening of adhesions to support higher intracellular tensions [34]. As osteoblasts possess typically larger cell morphologies, they require larger focal adhesions to support their cytoskeleton [35]. In agreement with this, the results demonstrate that focal adhesions of MSCs are formed on and around the NWs within the first days of culture (Fig. 2). This morphology then progresses to a thinner, elongated shape after one and two weeks, as focal adhesions grow (Fig. 3). Lastly, focal adhesions are further modulated to extend and become more polygonal while under magnetic field modulation of the NWs (Fig. 4). It has been evidenced that external mechanical forces strengthen the cell’s initial integrin-ECM adhesions into focal adhesion complexes through the recruitment of vinculin, which is absent from initial adhesions, resulting in focal adhesion elongation [36]. Indeed, Sniadecki and colleagues observed this phenomenon when applying a magnetic field (200 mT) to fibroblast cells cultured on microposts embedded with magnetic NWs, resulting in an increase in average cell focal adhesion area [37]. Interestingly, this effect was more pronounced for multiple magnetic stimulations. We believe a similar scenario plays out in the case of our magnetic NW scaffold, where the formation of focal adhesion points is probably initiated and guided by the NWs, which at later time points (i.e., 1–2 weeks) allow cell extension and focal adhesion growth. Such an effect is then enhanced under the mechanical modulation of the NWs when subjected to a magnetic field, leading to an earlier focal adhesion elongation and thus an earlier expression of the osteogenic marker OPN (Fig. 4).

### Osteogenic gene expression under a magnetic field

A combination of osteogenic markers is often used to evaluate osteogenesis progression [5, 38]. Therefore, we quantified OPN, Runx2, ALP, and OCN expression levels to determine if they were influenced by the NW topography and its magnetic field-mediated modulation. Generally, there are four principal developmental phases of gene expression during bone cell phenotype progression and specific genes are upregulated or downregulated at different times during this process [39]. After the initial developmental phase of cell proliferation, the process of osteoblast differentiation involves a cascade of cellular signaling pathways as cells progress into the ECM maturation phase. Pathways such as Wnt, BMP, and MAPK are believed to promote osteoblast differentiation by increasing Runx2 expression [40–42]. The transcription factor Runx2 is an early marker of osteogenic differentiation and regulates the expression of several bone-associated markers: OPN, OCN, ALP, and type-1 collagen. ALP is also an early and ubiquitous marker expressed by all osteoblasts, the expression of which peaks during the ECM maturation phase [43]. OPN is a regulator of the osteogenic commitment of MSCs whose expression begins in the proliferation phase and peaks in the third phase, marked by matrix mineralization. Meanwhile, OCN expression is mostly restricted to terminally differentiated osteoblasts [44, 45], with maximum expression observed in the matrix mineralization phase.

**Fig. 5:**
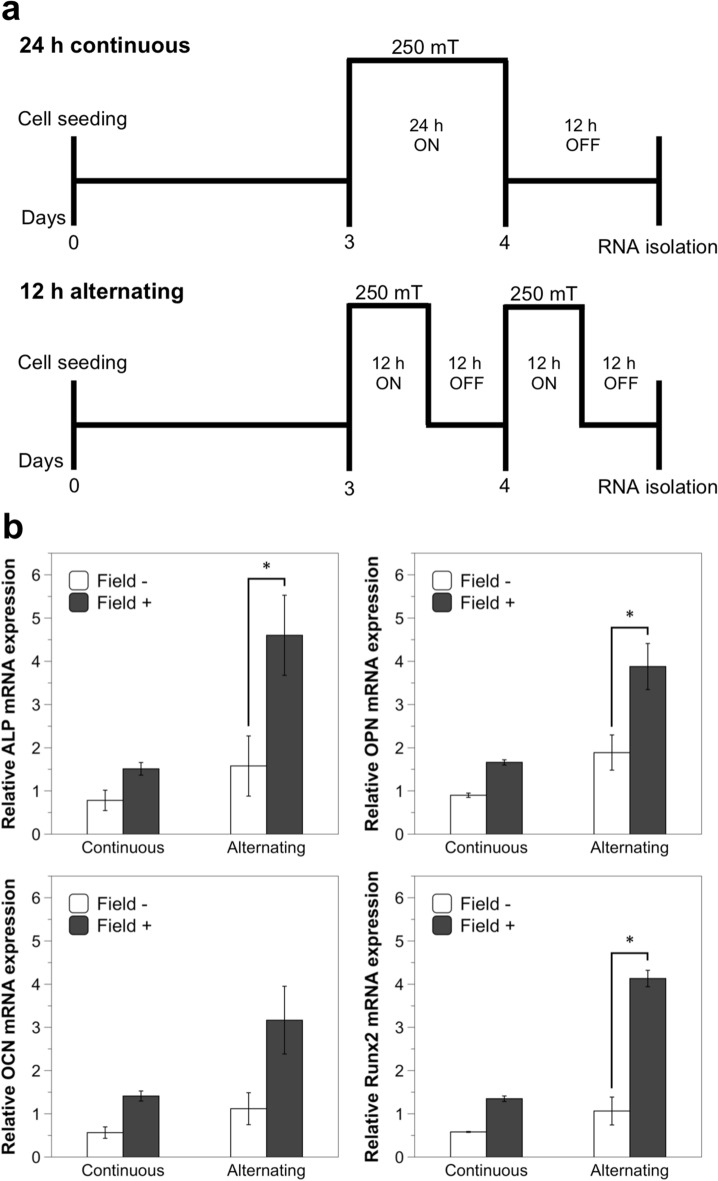
Relative gene expression of osteogenic differentiation markers in MSCs cultured on NWs with and without a magnetic field. **a** Magnetic field application profiles. A magnetic field with an intensity of 250 mT and a frequency of 0.1 Hz was applied in two different treatment profiles: 24 h (continuous) or 12 h per day (alternating). **b** Relative gene expression of ALP, OPN, OCN and Runx2 as determined by RT-PCR. Data represent the mean ± SEM (continuous: *n* = 2; alternating: *n* = 3, *p < 0.05).

Two different magnetic field application profiles were applied to MSCs on NWs: 24 h continuously or alternately 12 h on and off for 2 days (Fig. 5a). The magnetic field application resulted in an upregulation of osteogenic marker expression (Fig. 5b), which correlated with the previous OPN fluorescence observations. After 2 days of alternating magnetic field exposure (12 h a day), the mRNA expression levels of ALP, OPN and Runx2 were significantly elevated by 2.9, 2, and 3.9-fold, respectively, compared to those cultured on NWs without a magnetic field. The expression level of OCN, while 2.8-fold higher in cells under a magnetic field, was not found to be significant. A similar tendency also occurred in cells grown on the NW scaffold and continuously exposed to a magnetic field for 24 h; the expression levels of the four osteogenic markers were up-regulated compared to MSCs grown on the scaffold in the absence of a magnetic field, although not significantly. Overall, higher and significant upregulation of osteogenic markers was achieved with a magnetic field applied for 12 h a day over two days compared to a continuous single field application of 24 h. Finally, the expression of the early osteogenic markers ALP and Runx2 was observed at earlier culture time points in comparison to the usual 7–12 days [46, 47].

Osteoblast differentiation markers such as OPN and OCN are typically undetectable before day 12 and reach significant expression levels around day 16–20 under typical culture conditions [46]. In this research, they were enhanced as early as day two in MSCs cultured on the NW scaffold in the presence of a magnetic field. Additionally, there was an overall loss of CD105 and CD73 stem cell markers in cells grown on the NW scaffold under a magnetic field. Taken together, the results show that an application of a magnetic field promoted the osteogenic commitment of MSCs cultured on the NW scaffold within a relatively short timeframe.

As we previously discussed, focal adhesions play a critical role in the differentiation of MSCs through nanotopography-modulated mechanotransduction [48]. The formation of focal adhesions as MSCs come into contact with the ECM is a main driver of osteogenesis. This differentiation is regulated in part through the activation of focal adhesion kinase, an early marker of osteogenic commitment that stimulates the expression of ALP, OCN and Runx2 [49]. Indeed, an upregulation of focal adhesion kinase was reported for MSCs cultured on NWs, along with the upregulation of type 1 collagen and Runx2 osteogenic markers [18]. It was later proposed that the nanotopography of these NWs activates Ca^2+^ channels, as well as multiple mechanosensitive signaling pathways involved in cell adhesion and differentiation [50]. Other nanotopographies, such clusters of TiO_2_ nanotubes [51, 52], nanorods of TiO_2_ [53] or hydroxyapatite [54] also enhanced osteogenesis differentiation. For instance, TiO_2_ nanotubes induced integrin clustering, focal adhesion kinase phosphorylation, and the expression of OPN and OCN, whereas hydroxyapatite nanorods increased the expression of ALP, OPN, and OCN osteogenic markers, and mineral nodule formation. Thus, the increase in expression of ALP, OPN, OCN and Runx2 that we observe here is in alignment with the focal adhesion elongation observed due to the effects of the magnetic field, and could be explained as a mechanotransduction response of the nanotopography regulated by focal adhesions.

It should be stated that it remains a challenge to elucidate the mechanism by which the NW topography induces cell morphological changes, i.e., focal adhesion regulation. For instance, the modeling of the NW bending due to the magnetic force remains an initial limitation of this study. As can be observed on the scanning electron microscopy images of the NW scaffold, single NWs tend to aggregate at the tips, which could limit their single displacement. However, this could be an artifact of the drying process used for imaging, and as the scaffold was never allowed to dry during the experimental work with MSCs the real spatial interface between a cell and the NWs in a liquid medium remains elusive. Furthermore, the forces a cell can exert on the NWs, as well as the viscosity of the cell medium, could additionally hinder the effective force load a NW can exert on the cell. It is, thus, likely that the calculated NW tip deflection of *δ*_*B*_ = 100 nm may be overestimated. Nevertheless, the added differentiation enhancement under magnetic field application that was observed here does indeed point to a possible mechanical modulation of the nanotopography as the primary mediator of cell differentiation, given the NWs under magnetic field still exert a force on the cells. Despite the outlined limitations, and to the best of our knowledge, this is the first study that shows how a magnetoreceptive stem cell culture scaffold can increase the expression of osteogenic markers and initiate osteogenic commitment.

## Conclusion

In summary, we developed a scaffold for cell culture featuring a remotely modulated NW surface that promotes MSC differentiation. The nanotopography of the scaffold induced the expression of the osteogenic marker OPN after 1 week of culture. Modulation of the magnetoreceptive NW surface by the application of a low frequency magnetic field resulted in the onset of osteogenesis after only two days of culture, observed by the upregulation of OPN, ALC, OCN and Runx2 osteogenic markers. Overall, the use of a magnetoreceptive NW scaffold for the culture of MSCs significantly reduced the onset of osteogenic marker expression in the absence of the traditionally used biochemical methods. This effect can be significantly enhanced in the presence of a magnetic field, and we believe that it can be a result of nanotopography-mediated mechanotransduction.

The functionalities of the scaffold provide prospects for new tools in stem cell research and tissue engineering. Future work should particularly aim at understanding the mechanism of mechanotransduction of a NW-based nanosurface such as the one reported here, which remains a challenge. For instance, a thorough analysis of known cell mechanosensitive pathways such as focal adhesion kinase signaling, as well as cytoskeleton contractility regulation could provide an understanding of the manner in which cells transduce nanotopographical cues and how these may lead to the expression of cell differentiation markers. The studying of the expression of integrins associated to mechanosensing could also shed light onto the molecular pathways involved in the differentiation process. In addition, different cell spatial interfaces could be obtained by changes in the nanotopographical cues themselves, easily achievable through the fabrication process, such as NW length, spacing and diameter, which could in turn result in differential cell responses.

## Methods

### Fabrication of the nanowire scaffold

The fabrication procedure and the elemental and magnetic characterization of the Fe NW scaffold was previously described in detail by our group [25]. Briefly, nanoporous alumina templates were fabricated by a two-step anodization procedure using oxalic acid on a 99.9% aluminum disc substrate (2.5 cm in diameter). Following this, electrodeposition of Fe produced long aspect ratio NWs within the nanoporous alumina. Partial removal of the alumina through a wet etching process yielded a dense array of vertical NWs. The nanoporous alumina containing the dense array of NWs was used as the cell culture scaffold. The scaffold was never allowed to dry after the partial removal of the alumina to avoid NW collapse.

### Cell culture

Bone marrow derived human MSCs (Stemcell Technologies™) were grown in Mesencult MSC Basal Medium with Mesenchymal Stem Cells Stimulatory supplements (Stemcell Technologies™), as indicated by the vendor. The cells were kept in a 37 °C humidified incubator with 5% CO_2_. After reaching 80% confluence, cells were detached from the culture vessels using trypsin–EDTA and counted using the trypan blue staining method before further processing. Prior to cell seeding on the NW scaffold, the scaffold was thoroughly washed with 100% ethanol, phosphate-buffered saline (PBS), and cell medium. The aluminum substrates with the NW scaffold were placed in either 6- or 24-well plates or petri dishes for cell growth depending on the specific experimental analysis. MSCs between passages 2–5 were used for all experiments.

### Scanning electron microscopy imaging

All imaging was performed in a Quanta FEG600 (FEI) scanning electron microscope. For imaging of the free-standing network of NWs, the scaffold was subjected to critical point drying (Automegasamdri^®^-916B) to avoid NW collapse. For imaging of MSCs cultured on the scaffold, the cells were washed with PBS and fixed in 2.5% glutaraldehyde in 0.1 M cacodylate buffer for two hours at room temperature. The cells were thoroughly washed in the same buffer and then post-fixed in 1% osmium tetroxide (Electron Microscopy Sciences) in cacodylate buffer for one hour in the dark. After washing with deionized water, a series of dehydration steps with 10, 30, 50, 70, 90 and 100% ethanol were followed, at five minutes each. The sample was then dried at the critical point and sputter-coated with gold–palladium (5 nm) before imaging.

### Immunofluorescence imaging

After the desired time of growth, the cells were fixed in paraformaldehyde for 20 min at room temperature. The cells were then washed several times with PBS, permeabilized with 0.5% Triton X-100 (VWR) in PBS for three minutes, washed thoroughly with PBS and then blocked in 10% goat serum (Thermo Fisher Scientific) in PBS with 0.05% Tween-20 (Thermo Fisher Scientific) (PBST) for one hour at room temperature. The following primary antibodies were added in PBST: anti-osteopontin (ab8448, Abcam), 1:1000 dilution; anti-vinculin (ab18058, Abcam), 1:100 dilution; anti-CD105 (ab11414, Abcam), 1:1000 dilution or anti-CD73 (ab54217, Abcam), 1:1000 dilution, then incubated for one hour at room temperature in the dark. The cells were then washed with PBST and the corresponding secondary antibodies were added in PBST: Cy3-conjugated Goat anti-Rabbit IgG (A10520, Thermo Fisher Scientific), at a dilution of 1:500 and 1:250 for osteopontin and vinculin labeling, respectively; Alexa Fluor^®^ 488-conjugated Goat anti-Mouse IgG1 (A21121, Thermo Fisher Scientific), 1:250 dilution, or Alexa Fluor^®^ 594-conjugated Goat anti-Mouse IgG2a (A21135, Thermo Fisher Scientific), 1:250 dilution, then incubated for one hour at room temperature in the dark. After washing with PBS, F-actin was stained using 150 nM Alexa Fluor^®^ 488 phalloidin (Molecular Probes™) in PBS for 15 min at room temperature in the dark. The cells were washed with PBS before analysis under a Leica DMI6000 B inverted fluorescence microscope (the NW scaffold with cells were flipped for image acquisition). The ImageJ open software was used to quantify the cell area using the F-actin staining as a reference. For quantification of OPN, the corrected total cell fluorescence (CTCF) method was followed [55], with approximately 20 cells for each analysis. The area of each cell was multiplied by the mean background fluorescence, and then substracted from the integrated density using the ImageJ software.

### Magnetic field application

The application of a magnetic field to stem cells cultured on the magnetic NWs was performed with a projected field electromagnet (GMW 5201) coupled to a power supply (Agilent Technologies N5768A). The process was controlled using an in-house LabVIEW (National Instruments) code, allowing control over the frequency and current applied to the electromagnet. The NW scaffold with the cells was subjected to a magnetic field with an intensity of 250 mT at a frequency of 0.1 Hz for all experiments. For the immunofluorescence studies, the magnetic field was applied for 12 h per day. For the gene expression studies, the magnetic field was set either as continuous (24 h) or alternating (12 h per day). The magnetic field was applied in a parallel direction to the scaffold (perpendicular to the NWs).

### Real-time PCR analysis

The MSCs were seeded on the scaffold at a density of 5 × 10^4^ and then placed in a 24-well plate. After the desired cultured time and magnetic field application, the cells were detached using trypsin and a cell scraper, followed by cell counting. Total RNA was isolated from MSCs that were cultured on the NW scaffold with and without a magnetic field exposure using an RNAeasy^®^ Micro Kit (Qiagen), according to the manufacturer’s instructions. The RNA concentration was measured using a NanoDrop™ 2000 Spectrophotometer (Thermo Fisher Scientific). Total RNA was extracted and used for reverse transcription using a High-Capacity cDNA Reverse Transcription Kit (Applied Biosystems™). RT-PCR was performed with the Fast SYBR^®^ Green Master Mix kit (Applied Biosystems™) using cDNA libraries in a total reaction volume of 20 μl, with primers specific for ALP, Runx2 (Cbfa-1), OCN and OPN genes (Table 1). Gene expression was normalized to glyceraldehyde 3-phosphate dehydrogenase (GAPDH), and the relative gene expression was calculated via the comparative C_T_ method. All reactions were performed in technical and biological triplicates.

**Table 1:** PCR primer sequences

Gene	Primer Sequence (5′–3′)	Length (bp)
GAPDH (housekeeping)	F: ATGGGGAAGGTGAAGGTCG R: TAAAAGCAGCCCTGGTGACC	70
ALP	F: GGAACTCCTGACCCTTGACC R: TCCTGTTCAGCTCGTACTGC	86
Runx2 (Cbfa1)	F: TGGCAGTCACATGGCAGATTTC R: TGCTAAATTCTGCTTGGGTGGG	148
Osteocalcin	F: GGCAGCGAGGTAGTGAAGAG R: CTCACACACCTCCCTCCTG	102
Osteopontin	F: CAAACGCCGACCAAGGAAAA R: GGAGGCAAAAGCAAATCACTGC	60

### Statistical analysis

For osteopontin fluorescence, the statistical significance was quantified using the one-way analysis of variance using the MATLAB software (MathWorks, Inc.). For the gene expression analysis, the statistical significance was quantified using the student’s t-test using the MATLAB software. Statistical significance was considered for values of *p* < 0.01 or *p* < 0.05 vs. the specific control, depending on the experiment.

## Supplementary Information

**Additional file 1: Figure S1.** Immunofluorescence staining of CD105 and CD73 on MSCs cultured on Fe-NWs. MSCs were cultured on Fe-NWs or on tissue culture treated plastic (NC, negative control) for the indicated times (2 days or 1 week), and subsequently stained for the MSC stem cell markers, CD105 (green) and CD73 (red). These images are representative images of n = 2 independent experiments. **Figure S2.** Immunofluorescence staining of CD105 and CD73 on MSCs cultured on magnetically activated Fe NWs. MSCs were cultured on magnetically activated Fe-NWs or on tissue culture treated plastic (*NC* negative control) for the indicated times (2 days or 1 week), and subsequently stained for the MSC stem cell markers, CD105 (green) and CD73 (red). The NWs and NC were exposed to a magnetic field with an intensity of 250 mT and a frequency of 0.1 Hz that was applied for 12 h per day. These images are representative images of n = 2 independent experiments.

## Data Availability

The datasets used and analyzed for this work are available upon request to the corresponding author.
