# Supplementary material for: Modulated nanowire scaffold for highly efficient differentiation of mesenchymal stem cells
Source: J Nanobiotechnology. 2022 Jun 16;20:282. doi: 10.1186/s12951-022-01488-5 (PMC9202102; doi:10.1186/s12951-022-01488-5)
Supplement: Supplementary file 1 — Additional file 1: Figure S1. Immunofluorescence staining of CD105 and CD73 on MSCs cultured on Fe-NWs. MSCs were cultured on Fe-NWs or on tissue culture treated plastic (NC, negative control) for the indicated times (2 days or 1 week), and subsequently stained for the MSC stem cell markers, CD105 (green) and CD73 (red). These images are representative images of n = 2 independent experiments. Figure S2. Immunofluorescence staining of CD105 and CD73 on MSCs cultured on magnetically activated Fe NWs. MSCs were cultured on magnetically activated Fe-NWs or on tissue culture treated plastic (NC negative control) for the indicated times (2 days or 1 week), and subsequently stained for the MSC stem cell markers, CD105 (green) and CD73 (red). The NWs and NC were exposed to a magnetic field with an intensity of 250 mT and a frequency of 0.1 Hz that was applied for 12 h per day. These images are representative images of n = 2 independent experiments. [file 12951_2022_1488_MOESM1_ESM.docx]

Supplementary Information

**Modulated nanowire scaffold for highly efficient differentiation of mesenchymal stem cells**

**Jose E. Perez^1a^, Bashaer Bajaber^1a^, Nouf Alsharif^1^, Aldo I. Martínez-Banderas^1^, Niketan Patel^2^, Ainur Sharip^1^, Enzo Di Fabrizio^3^, Jasmeen Merzaban^1*^ and Jürgen Kosel^2,4*^**

^1^Boscience Program, Biological and Environmental Science and Engineering Division, King Abdullah University of Science and Technology (KAUST), Thuwal, 23955-6900, Kingdom of Saudi Arabia.

^2^Electric and Computer Engineering Program, Computer, Electrical and Mathematical Science and Engineering Division, King Abdullah University of Science and Technology, Thuwal 23955-6900, Kingdom of Saudi Arabia.

^3^DISAT Department, Corso Duca Degli Abruzzi. 24, 390110907336, 10129 Turin Politech, Italy.

^4^Division of Sensor Systems, Silicon Austria Labs, High Tech Campus Villach, A-9524 Villach, Austria.

^a^These authors contributed equally to this paper.

*Email: [jurgen.kosel@kaust.edu.sa](mailto:jurgen.kosel@kaust.edu.sa), [jasmeen.merzaban@kaust.edu.sa](mailto:jasmeen.merzaban@kaust.edu.sa)

**Nanowire Deflection**

The magnetic nanowire (NW) was modeled after an end loaded cantilever beam. A force in the form of a magnetic field is applied on one of the free ends of the beam (i.e., the NW), the latter of which thus experiences an elastic deflection defined as:

| $\delta_{B}=\frac{FL^{3}}{3EI},$ | (1) |
| --- | --- |

where *F* is the force of the magnetic field that is applied to the system, *L* is the length of the beam or NW, E is the elastic modulus of the material (*E* *=* 210 GPa for bulk Fe), and *I* is the moment of inertia, defined as:

| $I=\frac{\pi}{4}r^{4},$ | (2) |
| --- | --- |

where *r* *=* radius of the NW. The force *F* of the magnetic field that is applied to the free end of the NW in equation (1) was modeled as the equivalent point load, which is the total force applied to the beam divided by its length:

| $Equivalent point load=\frac{F}{L},$ | (3) |
| --- | --- |

with the force F corresponding to the magnetic torque of a single NW, the formula of which is:

| $\tau_{m}=M\pi r^{2}l\mu_{0}H,$ | (4) |
| --- | --- |

where *µ_0_H = B* and *M = M_S_*. The dimensions of the NWs were provided in our previous study, as well as the *M_S_* value of the array of NWs [1]. Thus, solving for equation (1) yields an elastic deflection of approximately *δ_B_ =* 100 nm, and an equivalent point load of 240 pN.

**CD105/CD73 Immunofluorescence Staining**

**
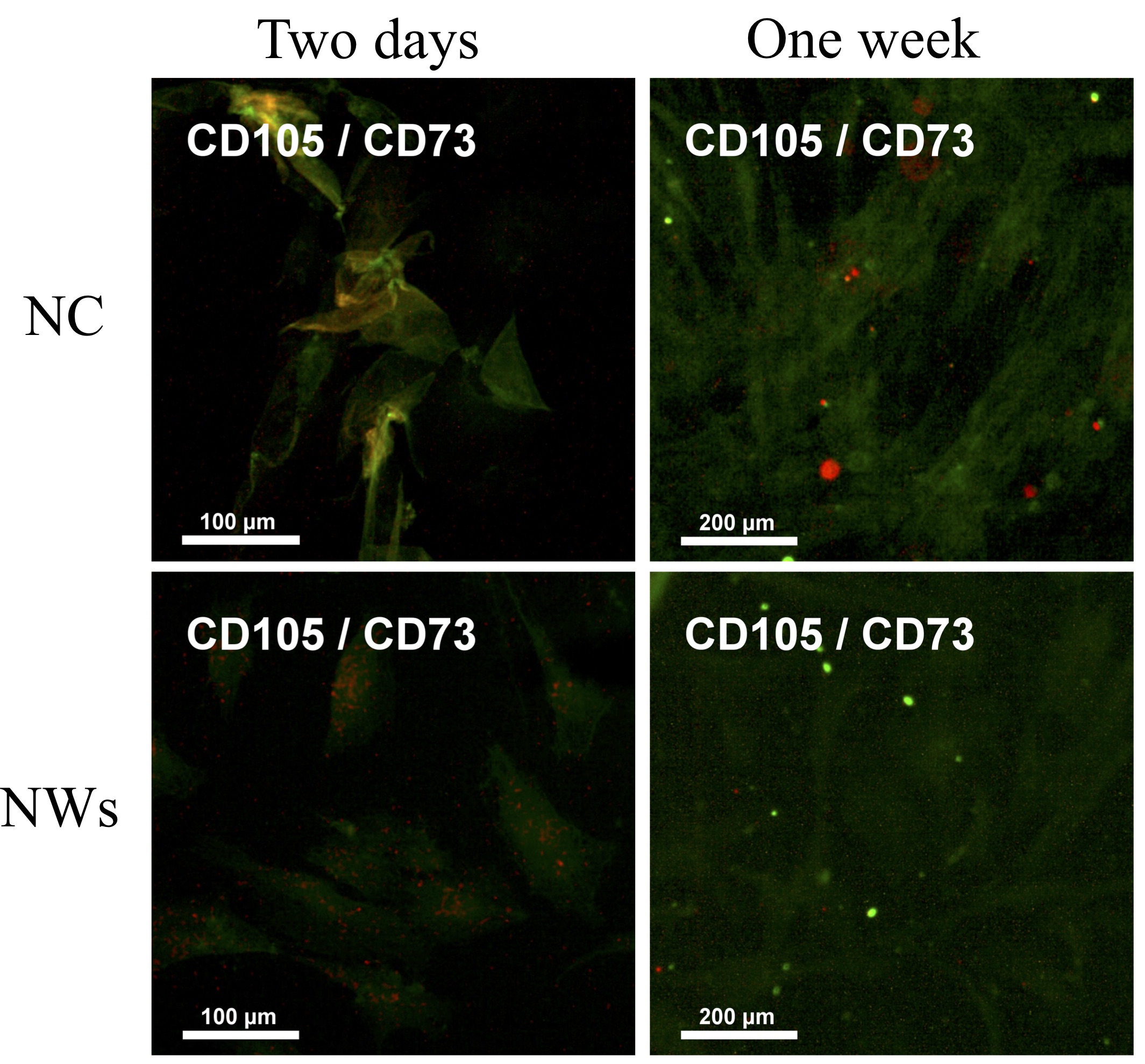
**

**Figure S1. Immunofluorescence staining of CD105 and CD73 on MSCs cultured on Fe-NWs**. MSCs were cultured on Fe-NWs or on tissue culture treated plastic (NC, negative control) for the indicated times (2 days or 1 week), and subsequently stained for the MSC stem cell markers, CD105 (green) and CD73 (red). These images are representative images of n=2 independent experiments.

**
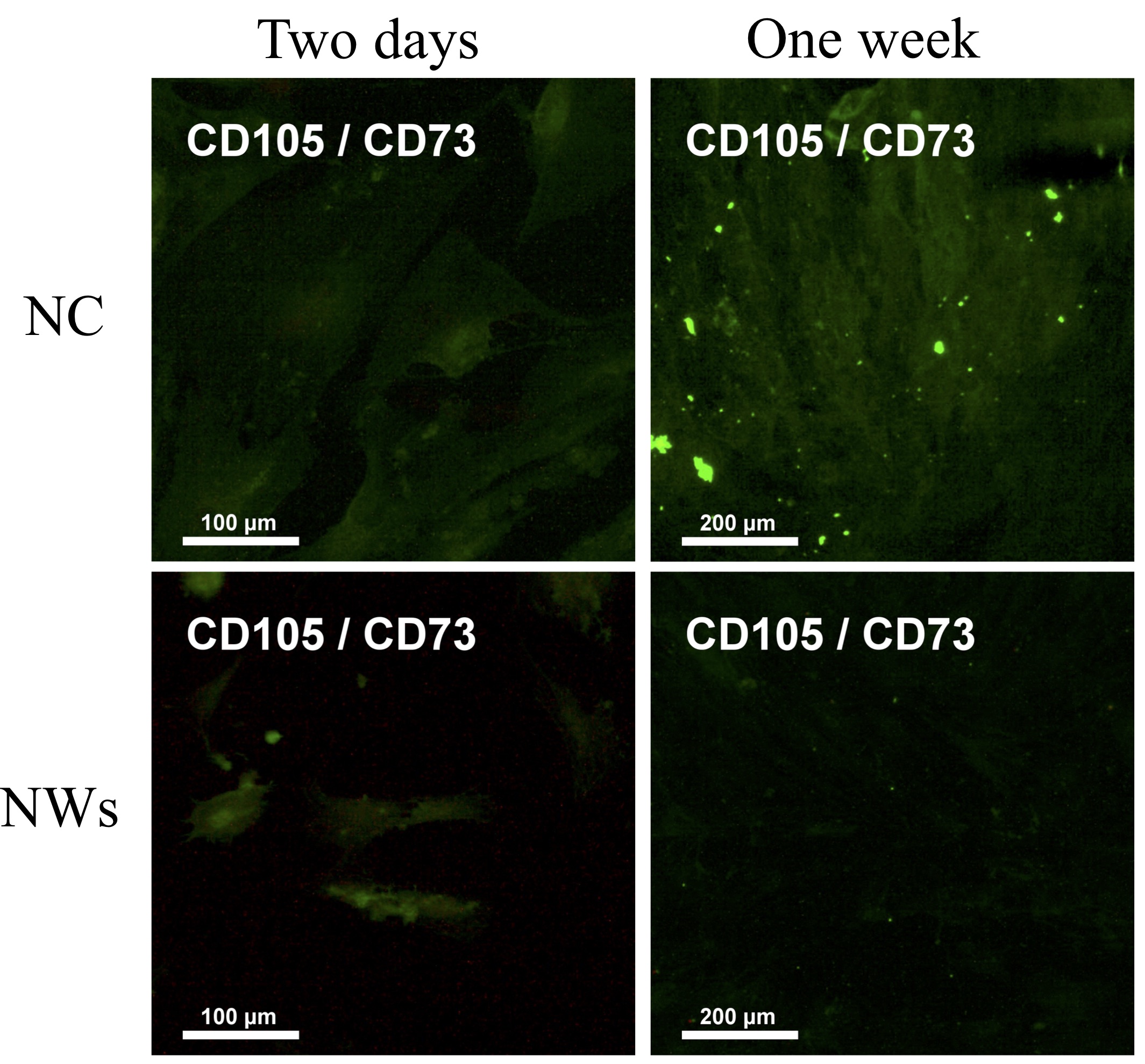
**

**Figure S2. Immunofluorescence staining of CD105 and CD73 on MSCs cultured on magnetically activated Fe NWs**. MSCs were cultured on magnetically activated Fe-NWs or on tissue culture treated plastic (NC, negative control) for the indicated times (2 days or 1 week), and subsequently stained for the MSC stem cell markers, CD105 (green) and CD73 (red). The NWs and NC were exposed to a magnetic field with an intensity of 250 mT and a frequency of 0.1 Hz that was applied for 12 hours per day. These images are representative images of n=2 independent experiments.

**References**

[1] Perez JE, Ravasi T, Kosel J. Mesenchymal stem cells cultured on magnetic nanowire substrates. Nanotechnology. 2017;28:55703–11.

[2] McCloy RA, Rogers S, Caldon CE, Lorca T, Castro A, Burgess A. Partial inhibition of Cdk1 in G2 phase overrides the SAC and decouples mitotic events. Cell Cycle. 2014;13:1400–12.
